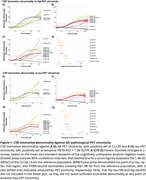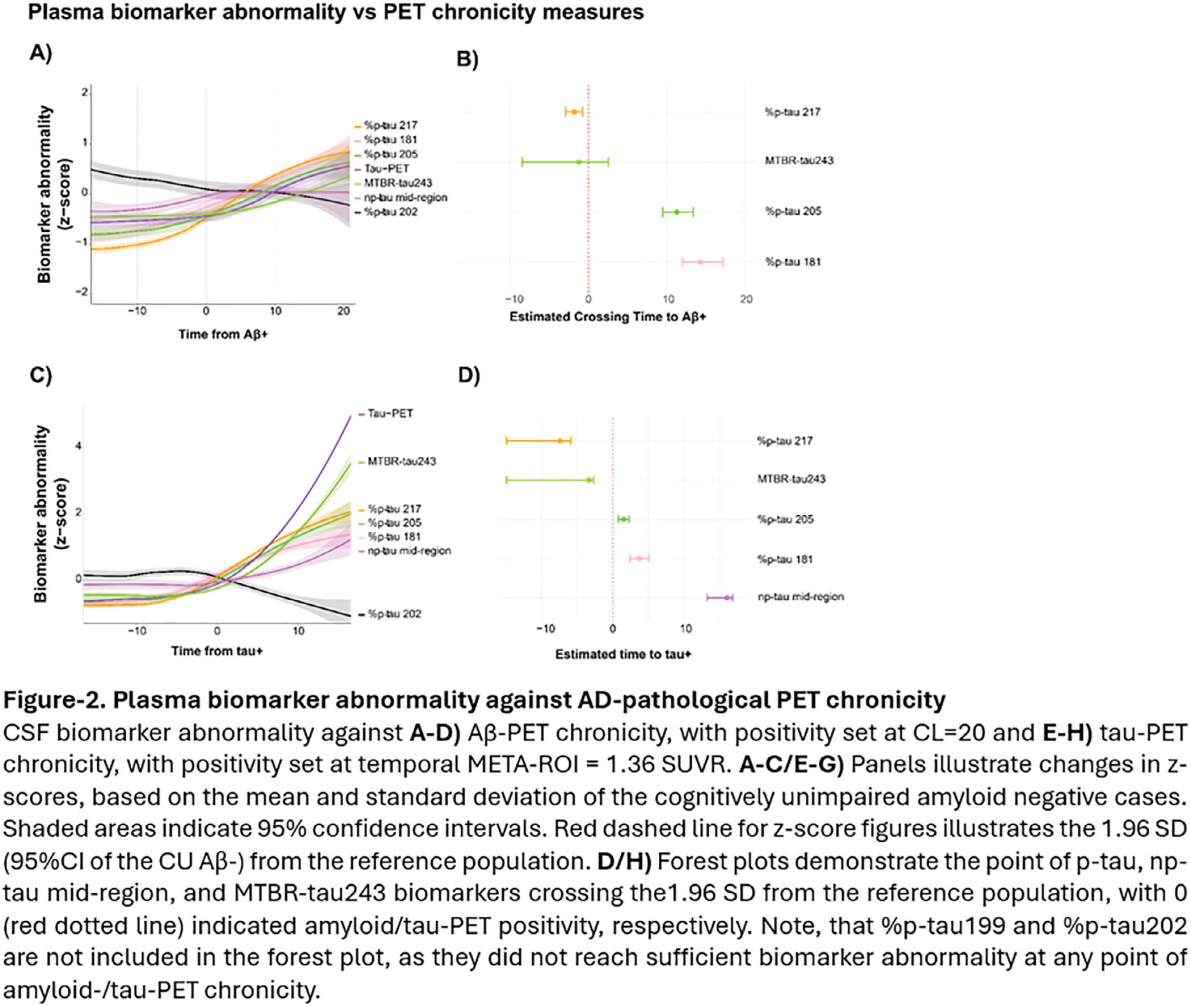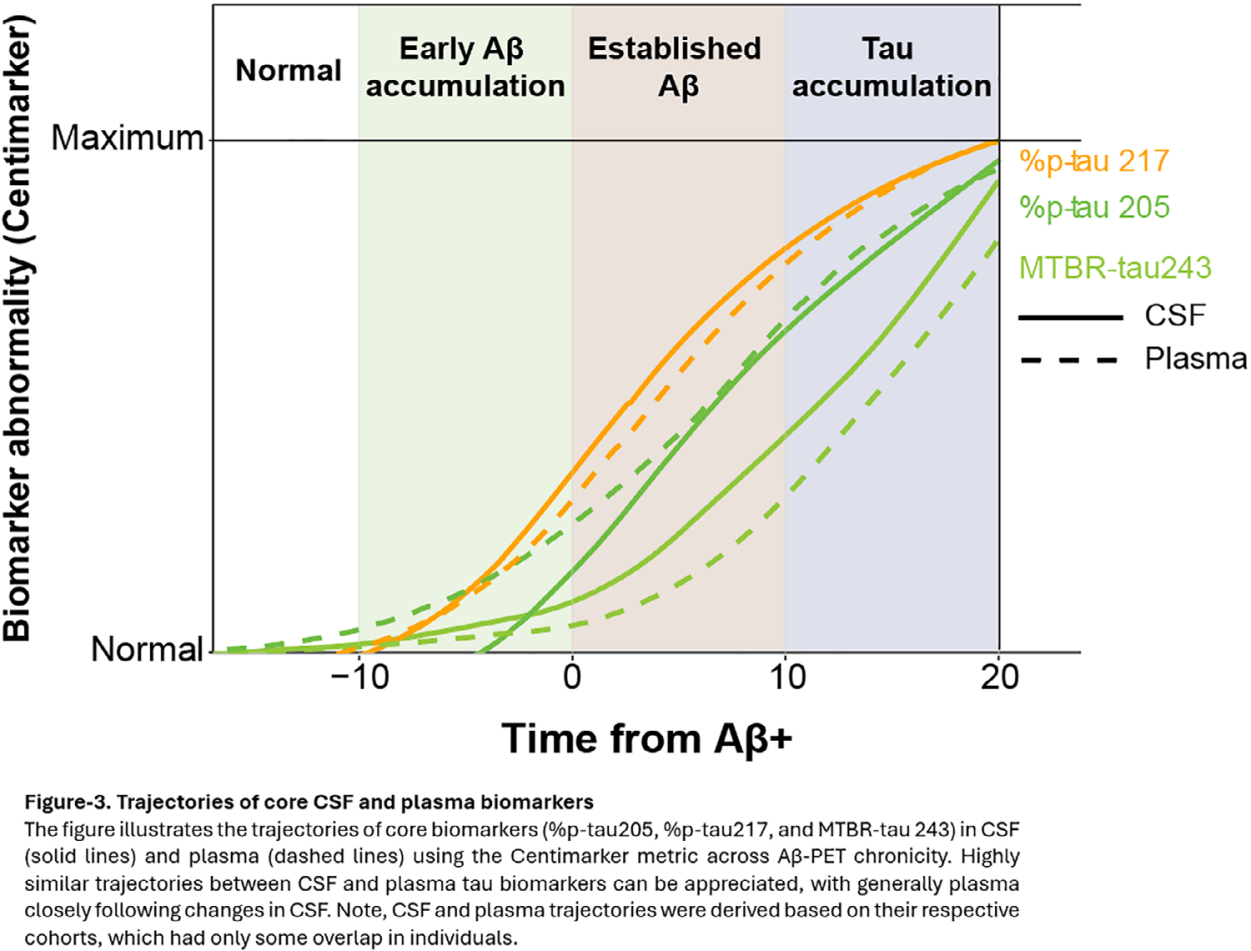# Trajectories of CSF and plasma MTBR‐tau 243 and phosphorylated‐tau species across the Alzheimer's disease continuum

**DOI:** 10.1002/alz70856_098557

**Published:** 2025-12-24

**Authors:** Lyduine E. Collij, Gemma Salvadó, Kanta Horie, Nicolas R. Barthélemy, Tobey J. Betthauser, Olof Strandberg, Ruben Smith, Sebastian Palmqvist, Suzanne E. Schindler, Rik Ossenkoppele, Shorena Janelidze, Niklas Mattsson‐Carlgren, Randall J. Bateman, Oskar Hansson

**Affiliations:** ^1^ Clinical Memory Research Unit, Department of Clinical Sciences Malmö, Faculty of Medicine, Lund University, Lund, Sweden; ^2^ Department of Radiology and Nuclear Medicine, Amsterdam UMC location VUmc, Amsterdam, Netherlands; ^3^ Amsterdam Neuroscience, Brain Imaging, Amsterdam, Netherlands; ^4^ Clinical Memory Research Unit, Lund University, Lund, Sweden; ^5^ Barcelonaβeta Brain Research Center (BBRC), Pasqual Maragall Foundation, Barcelona, Spain; ^6^ Deep Human Biology Learning (DHBL), Eisai Inc., Nutley, NJ, USA; ^7^ Hope Center for Neurological Disorders, Washington University School of Medicine, St. Louis, MO, USA; ^8^ Department of Neurology, Washington University in St. Louis School of Medicine, St. Louis, MO, USA; ^9^ University of Wisconsin‐Madison School of Medicine and Public Health, Madison, WI, USA; ^10^ Wisconsin Alzheimer's Disease Research Center, School of Medicine and Public Health, University of Wisconsin‐Madison, Madison, WI, USA; ^11^ Memory Clinic, Skåne University Hospital, Malmö, Skåne, Sweden; ^12^ Department of Neurology, Washington University School of Medicine, St. Louis, MO, USA; ^13^ Alzheimer Center Amsterdam, Neurology, Vrije Universiteit Amsterdam, Amsterdam UMC location VUmc, Amsterdam, Netherlands; ^14^ Clinical Memory Research Unit, Department of Clinical Sciences Malmö, Faculty of Medicine, Lund University, Sweden, Lund, Sweden; ^15^ Wallenberg Center for Molecular Medicine, Lund University, Lund, Sweden; ^16^ The Tracy Family SILQ Center, St. Louis, MO, USA; ^17^ Washington University School of Medicine, St. Louis, MO, USA

## Abstract

**Background:**

To efficiently implement Alzheimer disease (AD) cerebrospinal fluid (CSF) and plasma tau biomarkers, it is key to understand how they change over the course of disease progression. We therefore examined the temporal trajectories of mass spectrometry‐based measurements of microtubule‐binding region of tau (MTBR‐tau243) and tau phosphorylation occupancies (%p‐tau) in both CSF and plasma against disease time in relation to Aβ‐PET and tau‐PET positivity.

**Method:**

We included 784 participants with plasma and 446 with CSF biomarker data from the Swedish BioFINDER‐2, ranging from cognitively unimpaired (CU), subjective cognitive decline (SCD), mild cognitive impairment (MCI) or dementia. Aβ‐PET and tau‐PET chronicity (*i.e.*, disease duration) was derived using the sampled iterative local approximation (SILA) algorithm, with positivity defined as 20 Centiloid and 1.36 SUVR, respectively. All biomarkers and continuous tau‐PET burden in the temporal meta‐ROI and Braak regions were z‐scored based on the CU Aβ‐negative group (CSF: *n* = 97, plasma: *n* = 116). For CSF Aβ_42/40_, data were inverted such that higher z‐scores related to higher abnormality (for consistency across all biomarkers). For visualization purposes of the plasma biomarkers, all biomarkers and tau‐PET burden were also z‐scored based on the whole population, as plasma MTBR‐tau 243 has essentially zero variance in CU Aβ‐negative individuals.

**Result:**

The CSF and plasma cohorts were on average 71.4(±8.5) and 72.2 (±9.3) years of age, 49.1%/50.8% was female, and 263/453 (0.59%/51.8%) were cognitively impaired, respectively. In both CSF and plasma, only %p‐tau217 changed early, with significantly increased levels just prior to Aβ‐PET positivity (Figure 1A‐D, Figure 2A/B). Other *p*‐tau species, such as %p‐tau181, changed later in CSF and plasma, had smaller dynamic ranges, and earlier ceiling effects (Figure 1E‐G, Figure 2C/D). Changes in CSF and plasma %p‐tau205 were closely associated with tau‐PET positivity onset (Figure 1H, Figure 2C/D). MTBR‐tau243 trajectories, especially in plasma, were closely associated with trajectories of cortical tau‐PET burden (Figure 1C, Figure 2C). Finally, CSF and plasma non‐phosphorylated mid‐region tau could be a potential late‐stage biomarker.

**Conclusion:**

CSF or plasma *p*‐tau217, *p*‐tau205, and MTBR‐tau243 provide information about different biological events in the disease cascade (Figure 3), which can benefit clinical trials and patient management in clinical practice.